# Anoxic Biodegradation of Isosaccharinic Acids at Alkaline pH by Natural Microbial Communities

**DOI:** 10.1371/journal.pone.0137682

**Published:** 2015-09-14

**Authors:** Simon P. Rout, Christopher J. Charles, Charalampos Doulgeris, Alan J. McCarthy, Dave J. Rooks, J. Paul Loughnane, Andrew P. Laws, Paul N. Humphreys

**Affiliations:** 1 School of Applied Sciences, University of Huddersfield, Huddersfield, UK; 2 Microbiology Research Group, Institute of Integrative Biology, University of Liverpool, Liverpool, UK; Oak Ridge National Lab, UNITED STATES

## Abstract

One design concept for the long-term management of the UK’s intermediate level radioactive wastes (ILW) is disposal to a cementitious geological disposal facility (GDF). Under the alkaline (10.0<pH>13.0) anoxic conditions expected within a GDF, cellulosic wastes will undergo chemical hydrolysis. The resulting cellulose degradation products (CDP) are dominated by α- and β-isosaccharinic acids (ISA), which present an organic carbon source that may enable subsequent microbial colonisation of a GDF. Microcosms established from neutral, near-surface sediments demonstrated complete ISA degradation under methanogenic conditions up to pH 10.0. Degradation decreased as pH increased, with β-ISA fermentation more heavily influenced than α-ISA. This reduction in degradation rate was accompanied by a shift in microbial population away from organisms related to *Clostridium sporosphaeroides* to a more diverse Clostridial community. The increase in pH to 10.0 saw an increase in detection of *Alcaligenes aquatilis* and a dominance of hydrogenotrophic methanogens within the Archaeal population. Methane was generated up to pH 10.0 with acetate accumulation at higher pH values reflecting a reduced detection of acetoclastic methanogens. An increase in pH to 11.0 resulted in the accumulation of ISA, the absence of methanogenesis and the loss of biomass from the system. This study is the first to demonstrate methanogenesis from ISA by near surface microbial communities not previously exposed to these compounds up to and including pH 10.0.

## Introduction

The current strategy for the management of the UK’s intermediate-level radioactive waste (ILW) inventory is disposal to a Geological Disposal Facility (GDF) employing a multi-barrier system. One design option for the ILW disposal area of such a facility is the use of a cementitious based backfill that will allow the development of a saturated anoxic, hyper-alkaline environment favouring the retardation of key radionuclides [1]. After the closure of a GDF, the overall pH in the ILW disposal areas is expected to be as high as pH 13, with this value falling over timescales of 10^4^ to 10^5^ years to pH ~10 [1]. However, values of ca. pH 10 may also be experienced in the chemically disturbed zone surrounding the GDF, or in niches within the engineered barrier system.

As of 2010, the U.K. ILW inventory includes an estimated 2,000 tonnes of cellulosic materials [2] which under these prevailing conditions will be subject to alkaline hydrolysis [3]. This abiotic process generates a range of soluble cellulose degradation products (CDP) including: the α and β forms of isosaccharinic acid (ISA) (Fig A in S1 File); the α and β forms of metasaccharinic acid and a range of small organic molecules. In the case of hemicellulose, xylo-isosaccharinic (X-ISA) acid is formed [4]. Of these products, α-ISA has received considerable attention on account of its ability to complex and enhance the mobility of radionuclides including Pu, Th and Cs [5–7].

The construction and operational phases of a GDF provide an opportunity for microbial contamination and colonisation of the facility by microorganisms from the near-surface environment [8]. The impact of microbial activity on the performance of a GDF has received considerable international attention [8–11]. Biogeochemical evolution [12–14], gas generation [12, 15–17], complexation [3, 18] and the fate of carbon-14 bearing wastes [19, 20] are important issues. Within the ILW disposal area of a GDF, corrosion derived hydrogen [15] and cellulosic materials represent the dominant electron donors [11] available to drive these microbially mediated processes. However, the hyper-alkaline environment of such a facility is likely to prevent the establishment of broad scale microbial activity. Rather, microbial activity will be confined to low pH microsites within the facility and associated engineered disturbed zone. The ability of alkaline cellulose hydrolysis [3] to mobilise degradable carbon through the generation of soluble CDP, will play a pivotal role in the establishment of these hotspots of microbial activity. Consequently, the microbial degradation of CDP and associated ISA will have a significant impact on the ambient geochemistry of a GDF [14] and the migration of the radioelements therein.

Due to the conditions under which ISAs are generated, microorganisms found in circum-neutral, near surface environments should not encounter these carbon sources. However, the degradation of both stereoisomers of ISA under iron reducing, sulphate reducing and methanogenic conditions has been observed at near neutral pH (pH 7.5) by consortia not previously exposed to these carbon sources [21]. Aside from geological disposal of cellulose containing radioactive waste, CDPs are also generated in the Kraft paper pulping process. Microorganisms present in contaminated soils associated with these processes have been shown to degrade ISA under aerobic and anoxic conditions up to a pH of 9.5 [22–25]. This pH range for ISA degradation was extended up to pH 10.5 under denitrifying conditions using inocula taken from soda lakes and alkaline contaminated sites [26]. More recently, it has been shown that an alkaliphilic inoculum from a 100 year old, hyperalkaline, lime kiln waste site [27] where ISA is generated in-situ [28] was capable of ISA degradation under a range of conditions [28, 29] above pH 9.0.

This study investigates the ability of near-surface consortia from circum-neutral environments to adapt to alkaline conditions whilst utilising CDP as a sole carbon. The aim is to determine the ability of these microbial communities to adapt to the post closure environment of a GDF. The data generated inform our understanding of the potential impact of microbial activity on the biogeochemistry of a GDF and the associated safety assessments of such a facility.

## Methods

### Starting culture

Sediment samples were taken from the launch area of Leeds/Liverpool canal at the University of Huddersfield (SE 14890 16416). Samples were taken using a weighted sampler and stored under anoxic conditions in sealed plastic containers at room temperature; samples were transferred into microcosms within 14 days of collection. As a site has not yet been selected for any potential GDF in the UK, the sediment samples used can be regarded as a generic, diverse source of anoxic microbial consortia. Permission from the University of Huddersfield was acquired prior to sample collection.

### Preparation and analysis of cellulose degradation products (CDP)

CDP were prepared using the methods of Cowper *et al* [30] with standard laboratory paper tissue used as the cellulose source for degradation. Tissue (200g) was added to 1.8L of N_2_ flushed 0.1M NaOH and 10 gL^-1^ Ca(OH)_2_ in a pressure vessel. The headspace was flushed with N_2_ for 30 min, before being sealed and placed in an incubator at 80°C. After 30 days, the vessel was allowed to cool before the resultant liquor was filtered through a 0.2 μm (pore diameter) Millipore filter unit under a nitrogen atmosphere. Bottles of CDP were covered with aluminium foil to exclude light and stored in a nitrogen atmosphere. Tissue samples were subjected to forage fibre analysis in order to determine the hemicellulose and cellulose compositions [31, 32]; all sample materials were ground to ~1-2mm particle size prior to analysis.

### Microcosm operation and chemical analyses

The experiment consisted of three 500 mL, mesophilic (25°C) microcosms operated on a ‘batch feed’ cycle (weekly feed and waste cycle) within 1500 mL borosilicate vessels (Blusens, Herten, Germany). The sediment samples (200 mL) were homogenised prior to being diluted using the mineral media specified in BS14853 [33] to a volume of 450 mL. Each microcosm was then fed 50 mL of CDP on a weekly basis (7 day cycle) implementing a waste/feed cycle in which 50 mL of the volume was removed and replaced with CDP. pH was then adjusted accordingly using conc. HCl to a pH of 7.5 and once stable, the resulting suspension was split and made up to 500 mL using mineral media. The duplicate microcosm was then fed in the same manner as before, except that pH was increased incrementally (0.5 pH units every 2 weeks) to a pH of 9.5 over an 8 week period using 4M NaOH. Once stable at pH 9.5, the microcosm was split and again made up to a final volume of 500 mL. This duplicate microcosm was then maintained at a pH of 10 using a pH controller employing N_2_ flushed 4M NaOH.

Once stabilised, 6 mL samples were taken on a daily basis over 3 consecutive feeds. Samples were centrifuged at 9000 x *g* for 10 min, the supernatant filter-sterilised (0.45 μm pore diameter filter) and samples kept at 4°C prior to analysis. In addition, 0.9 mL of sample was added to 0.1 mL of phosphoric acid (85%) for GC analysis and frozen at -20°C. The presence and concentration of volatile fatty acids were determined using gas chromatography on a HP GC6890 (Hewlett Packard, UK). Acidified samples (1 μL) were passed through a HP-FFAP column (Agilent Technologies, Santa Clara, US) under the following conditions: initial temperature of 95°C for 2 min, followed by an increase to 140°C at a ramp rate of 10°C min^-1^ with no hold, followed by a second ramp to 200°C at a ramp rate of 40°C min^-1^ with a hold of 10 min, falling to a post run of 50°C. Total organic carbon (TOC) was determined using a Shimadzu TOC 5050A (Shimadzu UK Ltd, Manchester, UK). Isosaccharinic acid concentrations in both the α- and β- conformations were measured by high performance anion exchange chromatography using pulsed amperometric detection (HPAEC-PAD) on a Dionex 3000 ion chromatography system (Dionex, Camberly, UK) employing a Dionex Carbopac PA20 column (3 x 150 mm, 6.5 μm particle size) and eluting with aqueous sodium hydroxide (0.05 mol L^-1^) against a range of standards [34]. In order to identify recalcitrant organic carbon present within the CDP liquor, fractions were collected from the HPAEC-PAD. A mass was then obtained for the analyte using liquid chromatography linked to mass spectroscopy (LC-MS) using an Agilent LC 1290 employing an Agilent Eclipse plus C18 column (4.6 mm x 150 mm, 9.5 Å pore size) and eluting with an acetonitrile gradient (5%-100%) in 0.1% formic acid at a flow rate of 0.4 mL min^-1^ and MS 6530 (Agilent Technologies, Santa Clara, US) with jet stream source in negative ion mode.

The volume of gas produced was measured using Quick Scan 1.8c software and apparatus (Challenge Technology, Arkansas, US) with gas sensors for methane (BCP-CH_4_), carbon dioxide (BCP-CO_2_) and hydrogen (BCP-H_2_) connected to BACCom12 multiplexer utilising BACVis software (BlueSens gas sensor GmbH, Herten, Germany). Protein and carbohydrate analyses were carried out in accordance with previously described methods [35, 36]. In addition, smaller scale microcosms (total volume 50 mL) were prepared in triplicate and amended with 50 μg mL^-1^ chloramphenicol; these were sampled on a daily basis and total ISA concentration determined as above. These served as abiotic controls to eliminate the possibility of precipitation and sorption events.

### Carbon mass balance

Liquid phase carbon mass balance closure was calculated as a percentage of output carbon mass divided by input carbon mass (Eq 1):

$$\text{closure}\left(\%\right)=\frac{\;\text{carbon recovered}{\;}_{\text{(aq)}}\;[\sum C_{\text{out}}\text{]}\left(m\text{g}\right)}{\text{initial carbon}{\;}_{\text{(aq)}}\text{ [}\sum C_{\text{in}}]\;\left(m\text{g}\right)}\text{x 100}$$ (1)

Carbon recovered was calculated as the sum of the carbon present in the components analysed (Eq 2), where other organic carbon was determined as the difference between the total organic carbon content and the identified carbon content (Eq 3):

Carbon recovered (mg)=[∑α-ISA, β-ISA, acetic acid, other volatile fatty acids, inorganic carbon, other organic carbon] (mg) (2)

Other organic carbon(mg)=Total organic carbon−[∑α-ISA, β-ISA, acetic acid, other volatile fatty acids] (mg) (3)

The initial carbon was the total carbon measured at T_0_ using data acquired from the total organic carbon analyser (Shimadzu TOC5050A, Shimadzu UK Ltd, Manchester, UK).

### DNA extraction

Samples (50 mL) were centrifuged at 9000 x *g* for 20 min, before the pellet was re-suspended in 3 mL of sterile mineral media [33]. DNA was extracted using a modified version of a previously described method [37]. Sample (0.5 mL) was added to a sterile 2 mL tube containing: 0.5 g of glass beads, 0.5 mL of 5% CTAB/phosphate buffer (120 mM pH 8.0) and 0.5 mL of phenol/chloroform/isoamyl alcohol (25:24:1). This was bead beaten in a RiboLyser (Hybaid, Germany) for 30 s at 5.5 ms^-1^. Tubes were then centrifuged at 4°C for 5 min at 14,000 rpm, after which the aqueous top layer was removed and transferred to a new tube (~500 μL) and mixed with an equal volume of chloroform/isoamyl alcohol (24:1) to form an emulsion. After a second centrifugation step at 4°C for 5 min at 9000 x *g*, the top layer was extracted and transferred to a fresh sterile tube, mixed with 2 volumes of 40% polyethylene glycol 8000 (PEG) solution and incubated overnight at 4°C. Following incubation, the mixture was centrifuged at 18,000 x *g* for 10 min at 4°C, the supernatant removed and the pellet washed with 200 μL of 70% ice cold ethanol, before being removed and the pellet air dried for 20 min at 36°C. The pellet was then re-suspended in 30 μL of DNase free water. The presence of DNA was verified by electrophoresis of 5 μL of samples in a 1.0% tris-acetate-EDTA agarose gel with ethidium bromide staining. Nucleic acid concentration was determined using NanoDrop spectroscopy (Thermo Scientific, USA) before being diluted to a concentration of 5–10 ng μL^-1^.

### 16S rRNA Gene Sequencing

The Bacterial 16S rRNA gene was amplified using broad specificity bacterial primers pA (5’- AGAGTTTGATCCTGGCTAG-3’) and pH (5’- AAGGAGGTGATCCAGCCGCA-3’) [38], and a 660bp fragment of the Archaeal 16S rRNA gene was amplified using primers Af (5’- CCCTAYGGGGYGCASGAG-3’) and Ar (5’-GGGCATGCACYWCYTCTC-3’) [39] where Y (C or T); S (G or C); W (A or T). PCR reaction mixture contained 5 μL of purified DNA solution, 1.5 μL of each primer (10 pmol μL^-1^ concentration), and 25 μL of BIOMIX red master mix (BIOLINE, UK) made up to 50 μL volume with PCR grade water. The reaction mixture was then incubated at 94°C for 5 min, and then cycled 35 times through three steps: denaturing (94°C, 1 min), annealing (60°C, 1 min), primer extension (72°C, 1 min 30s). This was followed by a final extension step of 72°C for 5 mins. PCR was verified by electrophoresis of 5 μL samples of product in a 1.0% agarose TAE gel with ethidium bromide staining. The remaining 45 μL of product was isolated using electrophoresis in a 1.0% agarose TAE gel stained with ethidium bromide; bands were then excised from the gel and product purified using an ISOLATE PCR and gel kit (BIOLINE, UK).

PCR products were ligated into the standard cloning vector PGEM-T easy (Promega, US) and transformed into *E*.*coli* JM109 competent cells (Promega, US). Transformed cells were grown on LB agar containing 100 μg mL^-1^ ampicillin overlaid with 40 μL of 100mM IPTG and 40 μL of 40 mg mL^-1^ X-GAL in dimethylformamide for blue-white colour screening for 16 h at 37°C. Insert-containing colonies were sub-cultured to LB plates containing ampicillin/IPTG/X-GAL described previously and incubated for 24 h at 37°C. Colonies were then transferred to 96 well plates containing 150 mg mL^-1^ ampicillin and sequencing performed using Sanger sequencing technology (GATC Biotech, Germany), with inserts being amplified using M13 forward (5’-GTTTTCCCAGTCACGAC-3’) and reverse (5’-CAGGAAACAGCTATGAC-3’) primers and M13 forward primer used as a sequencing start point. The 16S rRNA gene sequences were grouped into Bacterial and Archaeal divisions. The sequences were then aligned using the alignment package MUSCLE [40]. Aligned sequences were then chimera checked using Mothur [41] and sequences analysed against the NCBI database using Basic Local Alignment Search Tool (MegaBLAST) utilising the 16S ribosomal RNA sequences for Bacteria and Archaea. Sequences were then placed into phylogenetic families based on the closest sequence from the MegaBLAST output.

### Nucleotide sequence accession numbers

The 16S rRNA sequence data have been submitted to GenBank under accession numbers KM999232 to KM999360 and KM999361 to KM999498

## Results

### CDP composition

**Fig 1:**
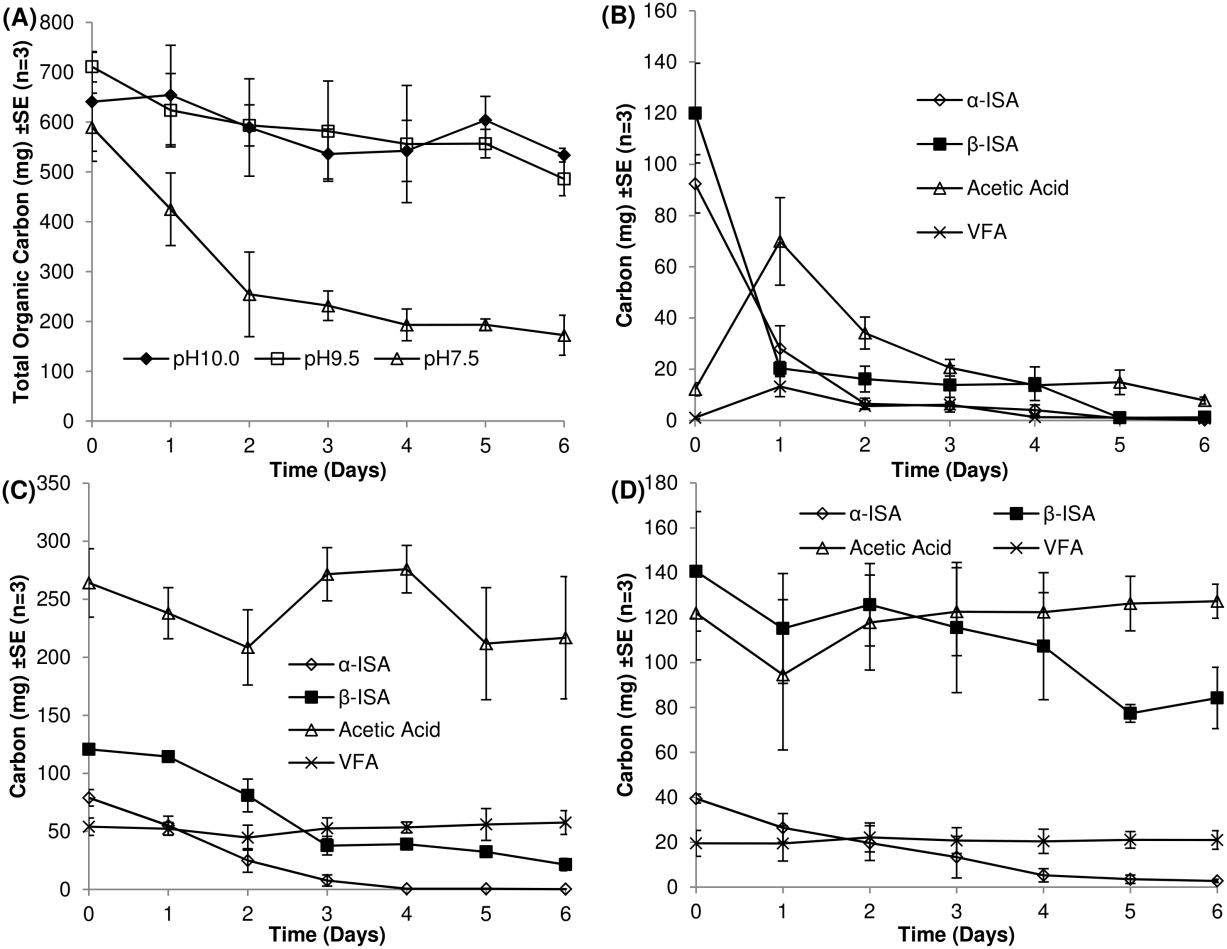
Removal of organic carbon from microcosms over 7 day sample period (A). Chemical analyses of microcosms operating at pH 7.5 (B), pH 9.5 (C) and pH 10.0 (D).

Forage fibre analysis indicated that the cellulose and hemicellulosic components constituted 161.8 g and 21.6 g of the original paper tissue respectively; post alkaline treatment, 59.3 g (36.7%) of cellulose and 1.75 g (8.1%) of the hemicellulose had been degraded. The resultant filtered liquor contained typically 3.54 ± 0.01 g L^-1^ total organic carbon of which 74% was contributed by the two stereoisomers of ISA in approximately equal quantities. Also present in the liquor were the α and β metasaccharinic acids and xylo-isosaccharinic acids resulting from the degradation of hemicellulose (6.6% of the total organic carbon)(Fig B in S1 File). Finally, formic, acetic, propionic and iso-valeric acids were present, together representing less than 1% of the total organic carbon combined (Fig 1).

In the control microcosms amended with chloramphenicol, removal of ISA was not observed across the three pH systems (Fig C in S1 File) indicating that removal of ISA in the test microcosms was microbially mediated and cannot be attributed to sorption or precipitation processes.

### Microcosm chemistry

**Fig 2:**
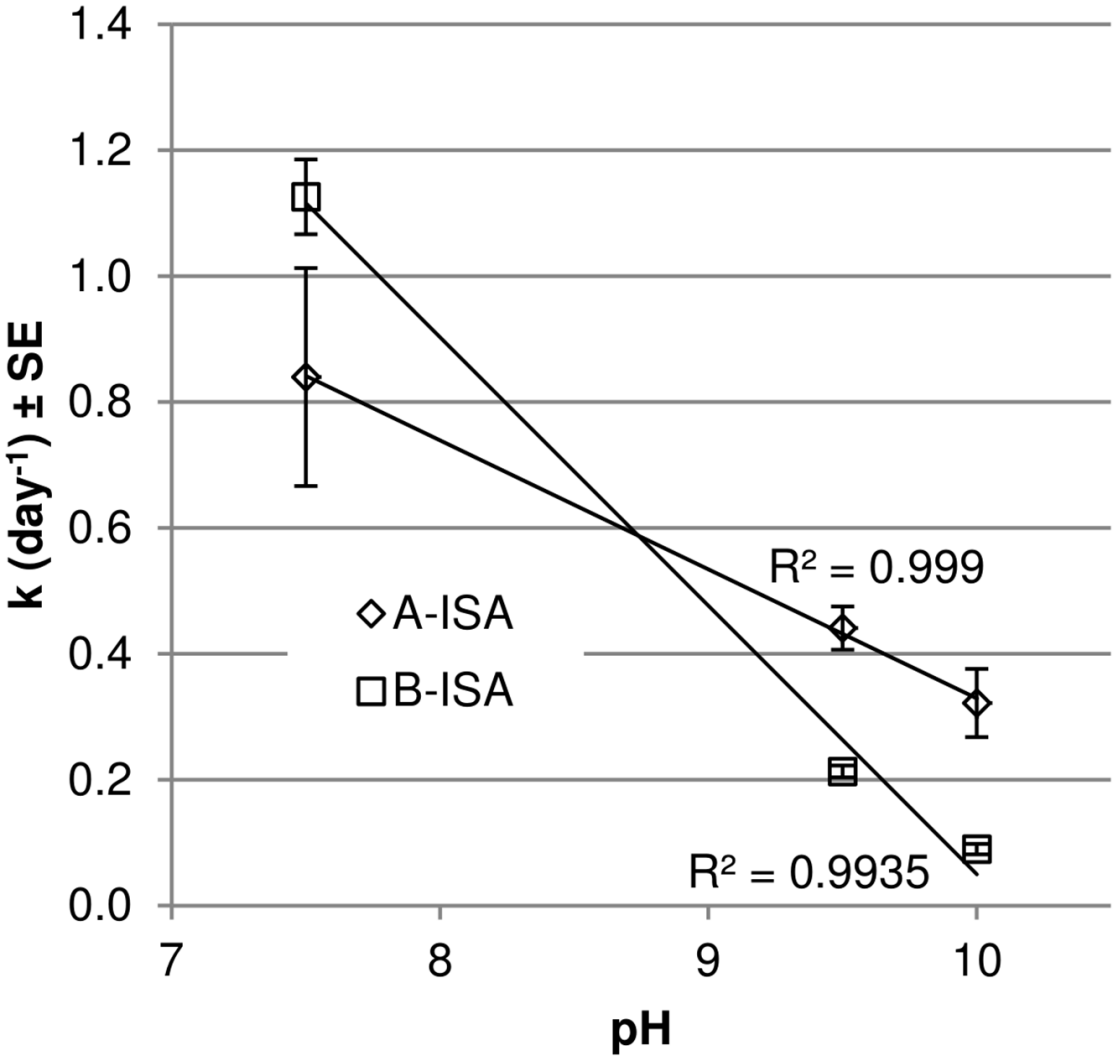
Rate of α and β ISA degradation at each pH system sampled. Mean values (n = 3) are presented ± SE.

At pH 7.5, both forms of ISA were completely removed within 7 days. As the pH increased to 9.5, α-ISA was completely removed from the system within the incubation period but at pH 10.0, 6.83 ± 1.82 mg of α-ISA remained. At both pH 9.5 and pH 10.0, β-ISA remained in the microcosm and appeared to be accumulating. The first order degradation rates of both forms of ISA are presented in Fig 2.

**Fig 3:**
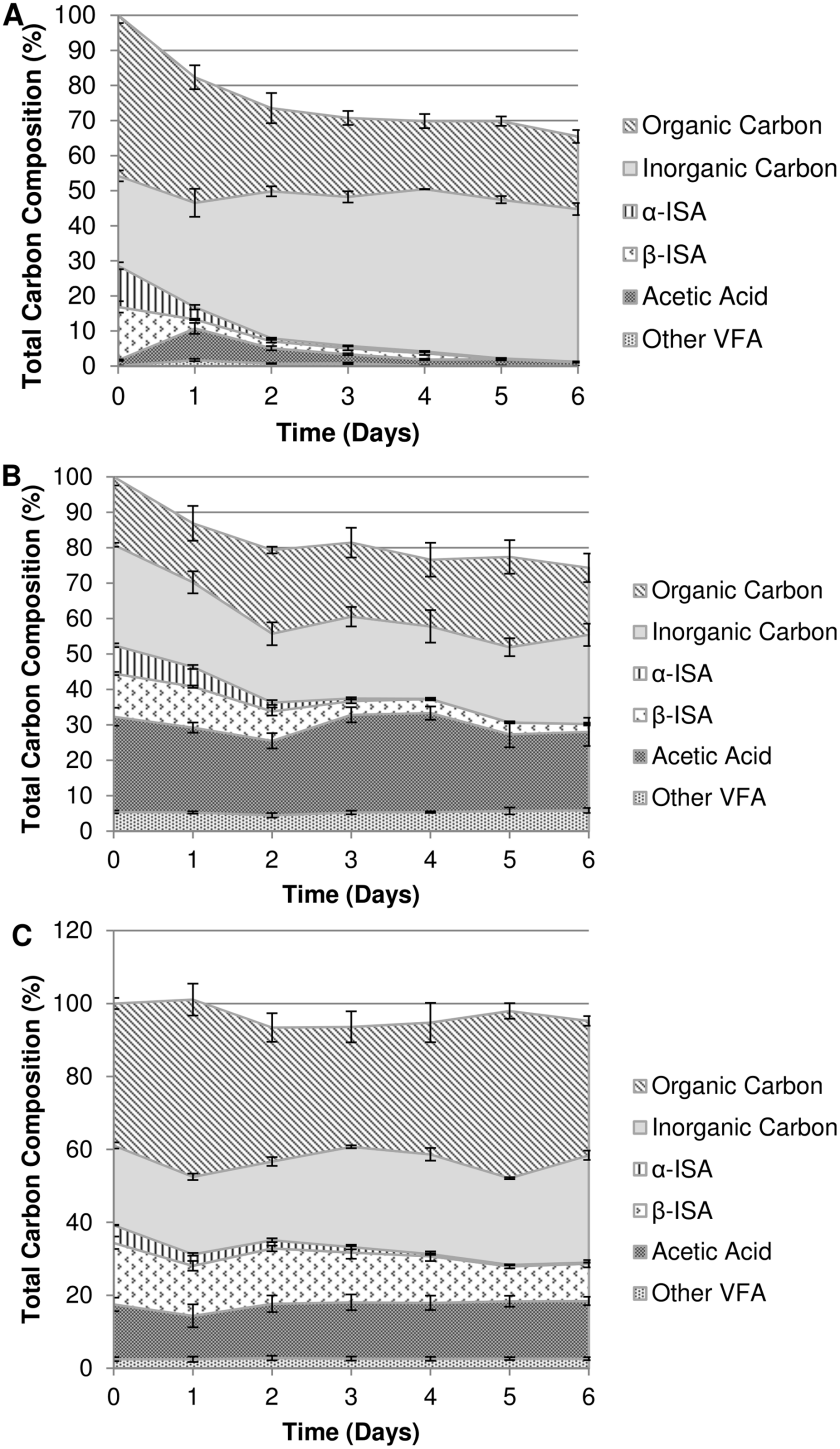
Liquid phase carbon mass balance profiles for pH7.5 (A), 9.5 (B) and 10.0 (C) microcosms. Mean values (n = 3) are presented ± SE.

The degradation rate of the α-ISA decreased from 8.40 x 10^−1^ day^-1^ to 4.41 x 10^−1^ day^-1^ between pH 7.5 and 9.5 and a further drop at pH 10.0 to 3.22 x 10^−1^ day^-1^. The degradation rate of the β form had reduced from 1.13 day^-1^ at pH 7.5 to 2.13 x 10^−1^ day^-1^ at pH 9.5. A further reduction in degradation rate was observed for β-ISA between pH 9.5 and 10.0, falling to 8.97 x 10^−2^ day^-1^. Fermentation processes were evident across all three microcosms through the generation of acetic acid as the most prevalent volatile fatty acid (VFA). Propionic, isobutyric, butyric and isovaleric acids were also generated across all three pH systems (data not shown). Acetic acid generated at pH 7.5 was gradually removed over the course of the experiment. At pH 9.5 and 10.0, acetic acid was generated but not completely removed from the system, and the dissolved carbon content was greater than that at pH 7.5 at the end of sampling. Liquid phase carbon mass balance closure can be seen in (Fig 3A–3C). At pH 7.5, closure stood at 65.4%, increasing to 74.3% at pH 9.5 and 95.2% in the pH 10.0 system.

**Fig 4:**
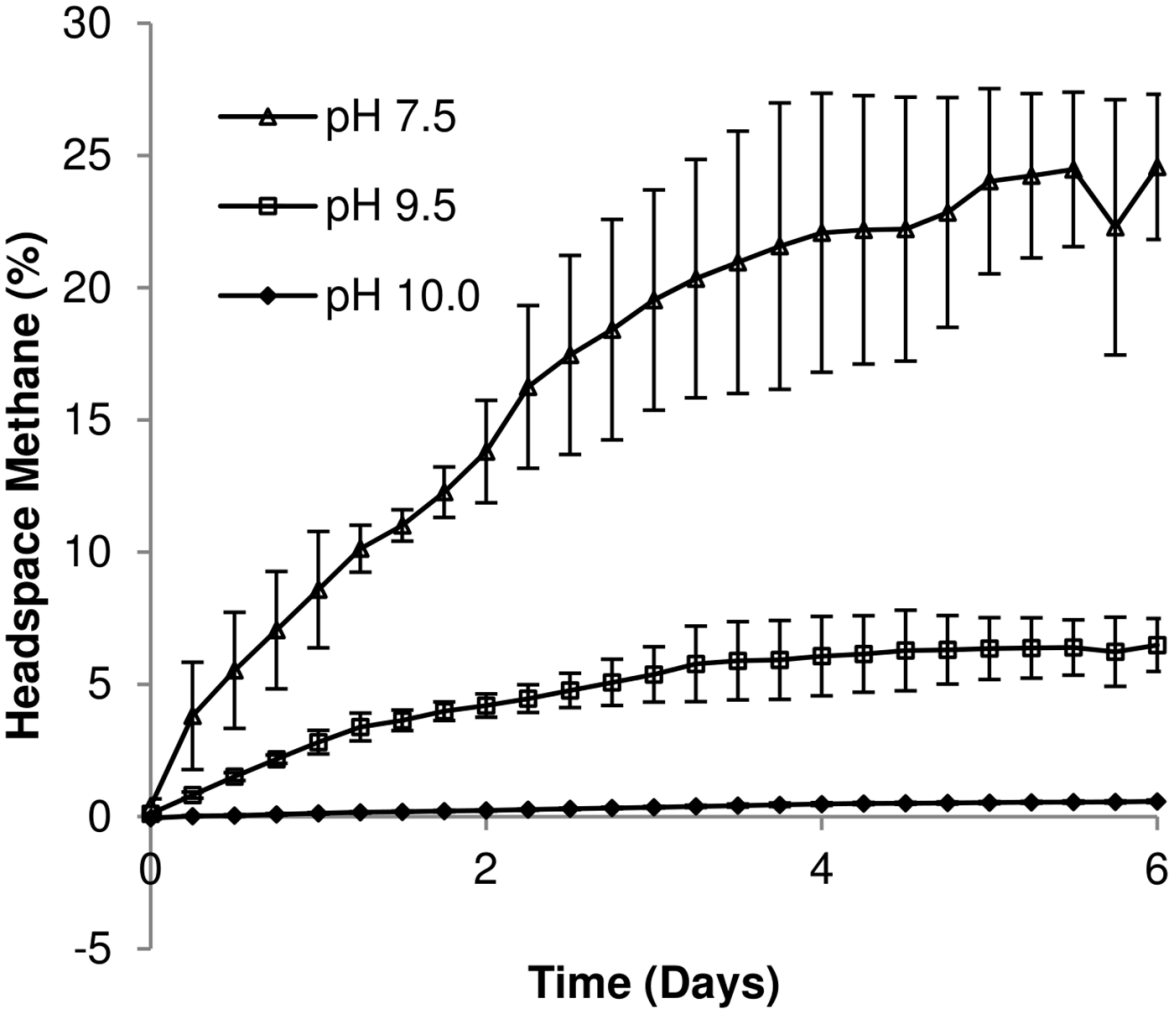
Methane gas evolution. Mean values (n = 3) are presented ± SE.

Protein and carbohydrate content (Figs D and E in S1 File) showed little variation throughout the 7 days of sampling. Methane gas evolved in each of the three microcosms (Fig 4), with methane as a greater percentage of the total gas generated in the pH 7.5 system; as pH increased, the volume of gas (data not shown) and percentage methane composition was also reduced. At the same time, the aqueous inorganic carbon content increased at pH 7.5, but the increase was less marked at pH 9.5 and 10.0, potentially due to the formation of carbonate precipitates.

### Bacterial 16S rRNA gene library

**Fig 5:**
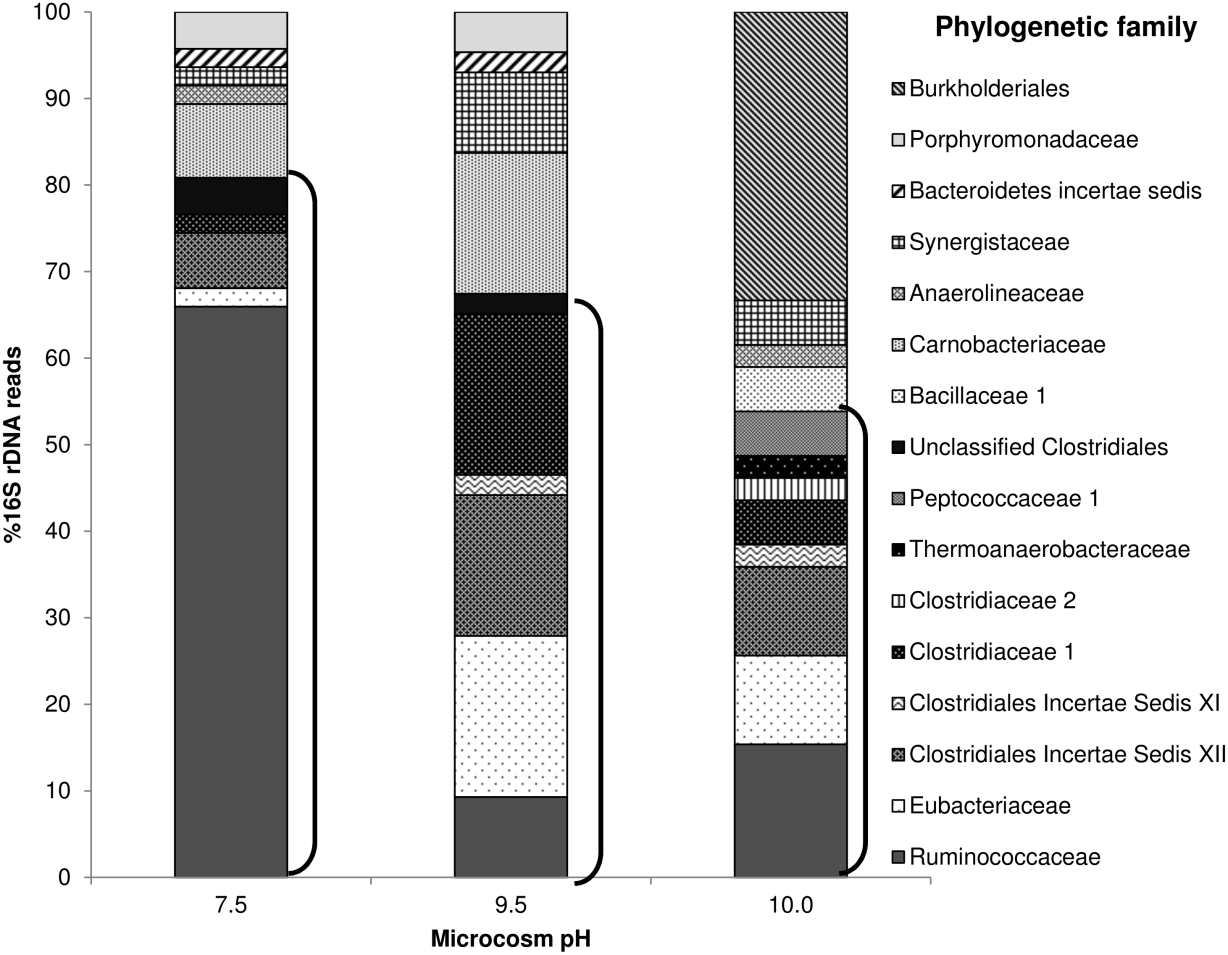
Eubacterial 16S rRNA gene clone libraries of CDP driven microcosms at pH7.5 (n = 47), 9.5 (n = 43) and 10.0 (n = 39). Clones were assigned to a family based on the closest sequence match obtained through MegaBLAST database search. Families associated to the group Clostridia are indicated by the black parentheses.

Closest sequence matches for the Bacterial clone library are presented in Table A in S1 File. The taxonomic composition of the 16S rRNA gene clone libraries of the three microcosms are compared in Fig 5. 47 Bacterial 16S rRNA gene sequences were obtained from the clone library from the pH 7.5 microcosm, of which 38 (79%) were associated with the phylogenetic class Clostridia (Fig 5). Within this class, 31 of the sequences belonged to the family Ruminococcaceae and 27 of these sequences were most closely related to *Clostridium sporosphaeroides* strain DSM 1294 (91–99% sequence similarity) when compared via the Blastn database. The pH 9.5 clone library was again dominated by Clostridia (67% of the 43 clones (Fig 5). Significantly, clones most closely matching *Clostridium sporosphaeroides* strain DSM 1294 were now completely absent from the clone library with only a total of four clones classified in the family Ruminococcaceae. At pH 9.5, within the Clostridia there was now a more even distribution of clones between the families Eubacteriaceae (8 sequences) *Clostridium* Insertae Sedis XII (7 sequences) and Clostridiaceae I (8 sequences). Clostridia sequences were further reduced in number in the pH 10.0 microcosm clone library but still represented 55% of the total, with the even distribution of clones observed at pH9.5 still evident (Fig 5). The most distinctive feature of the pH10.0 profile (Fig 5) appears within the non-Clostridia section of the clone library where *Alcaligenes aquatilis* strain LMG 22996 (98–99%, 12 sequences) from the family Burkholderiales was observed. Sequences affiliated with these organisms were not detected in the clone libraries from the pH7.5 and 9.5 microcosms.

### Archaeal 16S rRNA gene library

**Fig 6:**
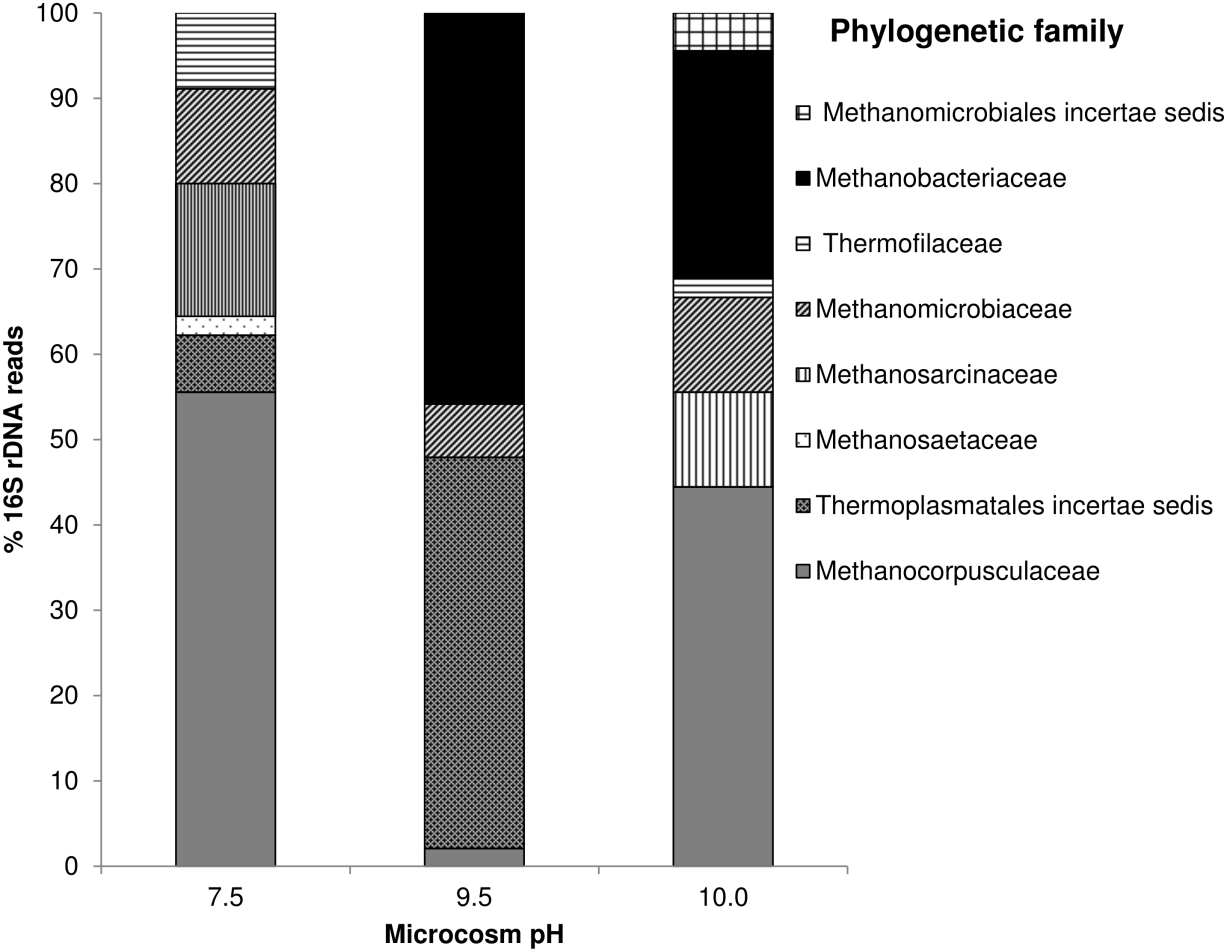
Archaeal 16S rRNA gene clone libraries of CDP driven microcosms at pH7.5 (n = 45), 9.5 (n = 48) and 10.0 (n = 45). Clones were assigned to a family based on the closest sequence match obtained through MegaBLAST database search strategy.

Closest sequence matches for the Archaeal clone library are presented in Table B in S1 File. The Archaeal clone libraries were dominated by methanogenic taxa (Fig 6). At pH 7.5, 25 (n = 45) of the sequences were found to match organisms from the family Methanocorpusculaceae, of which 23 sequences most closely matched *Methanocorpusculum aggregans* strain DSM 3027 (99% sequence similarity). The remaining sequences were spread across the families Methanosarcinaceae (7 sequences) and Methanomicrobiaceae (5 sequences), Thermoplasmatales insertae sedis (3 sequences), Thermofilaceae (4 sequences) and Methanosaetaceae (1 sequence). In the pH 9.5 microcosm, this profile had shifted significantly (Fig 6) with 46% (n = 48) of the clones most closely matching organisms from the family Methanobacteriaceae, and 17 of those sequences most closely matching *Methanobacterium flexile* strain GH (99% sequence similarity). A further 46% of the clones most closely matched organisms from the family Thermoplasmatales insertae sedis, all of which were most closely related to *Methanomassiliicoccus luminyensis* strain B10 (88–89% sequence similarity). The remaining clones were present in the Methanomicrobiaceae (3 sequences) and Methanocorpusculaceae (1 sequence) families (Fig 6). When the pH was increased to pH 10.0, the community structure again shifted such that sequences most closely associated with the family Methanocorpusculaceae predominated (44% n = 45), with sequences most closely matching *Methanocorpusculum aggregans* strain DSM 3027 (99% sequence similarity). The presence of sequences from the family Methanobacteriaceae had reduced to 27% of the total clones, with the closest species matches being *Methanobacterium alcaliphilum* strain NBRC 105226 (99%), *Methanobacterium flexile* strain GH (99%) and *Methanobacterium subterraneum* strain A8p (99%). The remaining sequences were distributed amongst the families Methanomicrobiaceae (5 sequences), Methanosarcinaceae (5 sequences), Methanomicrobiales insertae sedis (2 sequences), and Thermofilaceae (1 sequence) (Fig 6).

## Discussion

Although the α- and β- forms of isosaccharinic acid are not naturally encountered in the wider environment, bacteria that inhabit anoxic sediments are clearly capable of degrading these compounds through a fermentative, methanogenic pathway up to a pH of 10.0. As such, this is the first report of methanogenesis from ISA’s at pH > 9.5 from non-alkaliphilic consortia. Following an increase in pH to 11.0, ISA fermentation ceased and ISA accumulated in the system to theoretical values following subsequent feeding cycles (Fig F in S1 File). As expected, pH was an important rate limiting parameter, leading to the relative persistence of β-ISA in the system and the accumulation of acetic acid at pH 9.5 and 10.0. At these pH values, quantities of non-acetic volatile fatty acids were greater than those observed at pH 7.5. In the microcosms operating at elevated pH (9.5 and 10.0), a greater portion of organic carbon remained in the microcosm and consequently, increased aqueous carbon closure at higher pH is most likely due to the reduced generation of biomass and biogas within the systems. Whilst a portion of the organic carbon present in all the reactors was recalcitrant, one of the components within the original CDP feedstock (peak 7, S2 Fig) accumulated and remained un-degraded at elevated pH. This compound was identified as a C-8 octanedioic acid derivative, potentially formed from the condensation of two smaller molecules, as observed previously [42]. The accumulation of a C-8 octanedioic acid derivative could be significant should it exhibit any radionuclide complexation ability, particularly if its recalcitrance is common at high pH.

In these microbial consortia, clostridia appear to drive the metabolism of ISA to common fermentation end products allowing electron and carbon flow within these environments. At pH 7.5, organisms most closely related to *Clostridium sporosphaeroides* predominated, an observation in line with their documented ability to degrade cellulose [43, 44]. Within the microcosms operating at pH 9.5 and 10.0, the Clostridia were also prevalent but *Clostridium sporosphaeroides* and relatives were absent suggesting that although they have a broad substrate range [45, 46], they are not alkali tolerant. The microcosm population at pH 9.5 shifted away from the cellulosic Clostridia towards organisms known to be alkaliphilic, such as *Acidaminobacter hydrogenoformans* and *Alkalibacter saccharofermentans* [47, 48]. Organisms most closely matching these two species together with *Youngibacter multivorans* dominated (>50%) the pH 9.5 microcosm clone library (Fig 5 and Table A in S1 File), enriched from the pH 7.5 population where they were present as minor constituents *Y*. *multivorans* and *A*. *saccharofermentans* are known to ferment carbohydrates [48, 49], and may be the primary degraders of CDP here. *A*. *hydrogenoformans*, which is more commonly associated with the fermentation of amino acids [50] may be contributing to the cycling of dead microbial biomass (Fig 3), resulting from the increase in pH.

At pH 10.0 *A*. *hydrogenoformans* and *A*. *saccharofermentans* remained the dominant Clostridia within the clone libraries, however the prevalence of *Y*. *multivorans* was reduced, perhaps reflecting its reported pH maximum of pH 8.0 [49]. The number of species detected in the pH 10.0 microcosm increased to 17 from 14 at pH 7.5, with Clostridia still predominant. Among the species that were previously undetected was *Alcaligenes aquatilis*, which significantly made up 31.6% of the sequences in the clone library. These strains are capable of anaerobic growth and are associated with nitrogen cycling [51], suggesting that they are responding to an increased level of biomass turnover at elevated pH. The cessation of microbial activity at pH 11 may be linked to the absence of more recognised alkaliphilic Clostridia such as *Alkaliphilus* sp, which have previously been associated with ISA degrading consortia at pH11.0 [28]. The absence of *Alkaliphilus* sp may also be responsible for the lower overall rate of ISA degradation observed at both pH 9.5 and 10.0 when compared to those at pH11.0 [28].

Within the Archaeal clone libraries, hydrogenotrophic methanogens predominated across all three pH values. Acetoclastic methanogens were less abundant (24% at pH 7.5, 0% at pH 9.5 and 13.3% at pH 10.0) which is reflected in the chemical profiles of the microcosms where acetate was degraded completely at pH 7.5 (Fig 1B) but accumulated at more alkaline pH values (9.5, 10, Fig 1C and 1D). This acetate accumulation correlated with a reduction in the number of sequences affiliated to the acetoclastic *Methanosarcina* spp, which have a reported pH maxima of pH 8.5 [52–54]. This accumulation of acetate was non-stoichiometric and previous authors have suggested that some hydrogenotrophic methanogens, including *M*. *alcaliphilum* and *M*. *subterraneum*, assimilate acetate as a growth factor, rather than as a carbon source for methanogenesis [54, 55]. Reduced methane production at high pH from acetate was also observed by previous authors utilising soda lake consortia as starting inocula for bioreactors up to pH 10 [56]. Whilst the increase in pH had a negative impact on acetoclastic methanogens, the increase in pH to 9.5 resulted in an increased detection of *Methanobacterium* strains, all of which have been previously associated with hydrogenotrophic methanogenesis at elevated pH values [55, 57]. The presence of *M*. *alcaliphilum* was also observed within an alkaliphilic consortia cultured previously [28], suggesting that the pH range of these organisms is wider in mixed cultures than pure isolates [58].

The accumulation of acetic acid at elevated pH had no discernable impact on the rate of either α- or β- ISA degradation in subsequent feeds, however acid accumulation may have implications for pH buffering within the local geochemical environment as a result of microbial activity, which may in itself affect the solubility of uranium [59]. In a similar fashion, localised decreases in pH could affect the solubility of precipitated C-14 bearing carbonates, allowing hydrogenotrophic methanogenesis to generate C-14 bearing methane which could increase the concentration of C-14 in a bulk gas phase that may migrate from a GDF [19]. The impact of acetic acid accumulation on ambient pH may be attenuated below pH 9.5 by the re-establishment of acetic acid degrading microbial communities at these lower pH values.

In summary, this work is the first to have demonstrated that near surface microbial communities are capable of generating methane from the products of anaerobic alkaline cellulose degradation, i.e. ISAs, up to pH 10.0. Although, adaption to alkaline pH (≤10) was observed within a short timescale when compared to those expected for geological disposal, these results indicate that near surface microbial communities from circum neutral environments will be confined to niches where the pH is ≤10.0 unless further adaption occurs. In the absence of further adaption, the activity of these communities will be severely constrained by the ambient pH of a GDF which is expected to be >pH 11.0 for considerable periods of time [1]. A key constraint appears to be the presence of organisms from the genus *Alkaliphilus* within the consortia; their absence confines the fermentation of ISAs to a pH of ≤10.0. Members of this genus have been detected in anthropogenic hyper alkaline sites [27, 60] where in-situ ISA formation has been observed [28] and in natural hyperalkaline systems[61]. As pH increases methane generation becomes confined to the hydrogenotrophic pathway due to the loss of acetoclastic methanogens, resulting in the accumulation of acetic acid. The degradation rates for α and β ISA reported here are the first to be described at pH 9.5 and 10 for mixed communities under methanogenic conditions. The slower rates of β-ISA degradation relative to the α-form may hold particular significance, indicating the potential for β-ISA to remain available for radionuclide complexation over longer timescales than α-ISA.

## Supporting Information

S1 File Fig A: Alpha (L) and beta (R) conformations of isosaccharinic acid; Fig B: High performance anion exchange chromatography trace of CDP liquor.; Fig C: Total ISA concentration in control reactors amended with 50mgml^-1^ chloramphenicol; Fig D: Bradford assayed protein levels across all three systems.; Fig E: Total carbohydrate assay; Fig F: ISA and ATP assays of pH11 microcosm. Measured ISA concentration and relative light units (RLU) in first 12 weeks of sampling. Modelled ISA concentration shown for comparison.; Table A: Eubacterial clone libraries of pH 7.5, 9.5 and 10 microcosms, with the closest sequence match within the MEGAblast database; Table B Archaeal clone libraries of pH 7.5, 9.5 and 10 microcosms, with the closest sequence match within the MEGAblast database.
(DOCX)
