# Supplementary material for: Anoxic Biodegradation of Isosaccharinic Acids at Alkaline pH by Natural Microbial Communities
Source: PLoS One. 2015 Sep 14;10(9):e0137682. doi: 10.1371/journal.pone.0137682 (PMC4569480; doi:10.1371/journal.pone.0137682)
Supplement: S1 File — Fig A: Alpha (L) and beta (R) conformations of isosaccharinic acid; Fig B: High performance anion exchange chromatography trace of CDP liquor.; Fig C: Total ISA concentration in control reactors amended with 50mgml-1 chloramphenicol; Fig D: Bradford assayed protein levels across all three systems.; Fig E: Total carbohydrate assay; Fig F: ISA and ATP assays of pH11 microcosm. Measured ISA concentration and relative light units (RLU) in first 12 weeks of sampling. Modelled ISA concentration shown for comparison.; Table A: Eubacterial clone libraries of pH 7.5, 9.5 and 10 microcosms, with the closest sequence match within the MEGAblast database; Table B Archaeal clone libraries of pH 7.5, 9.5 and 10 microcosms, with the closest sequence match within the MEGAblast database. (DOCX) [file pone.0137682.s001.docx]

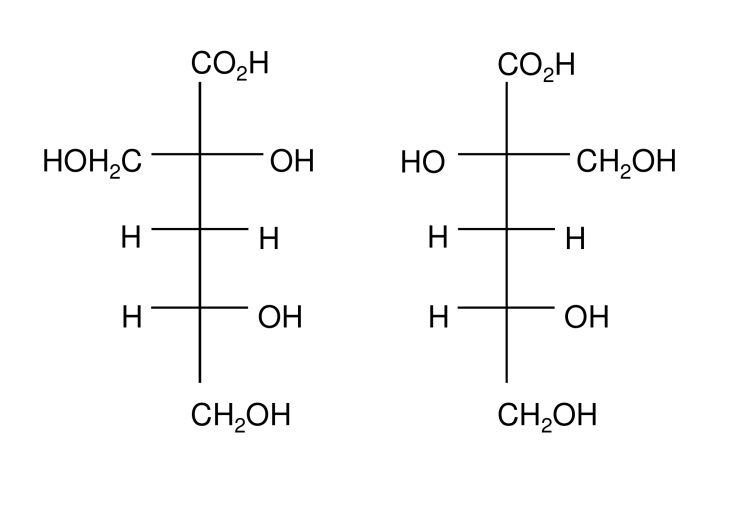


Figure A. Alpha (L) and beta (R) conformations of isosaccharinic acid


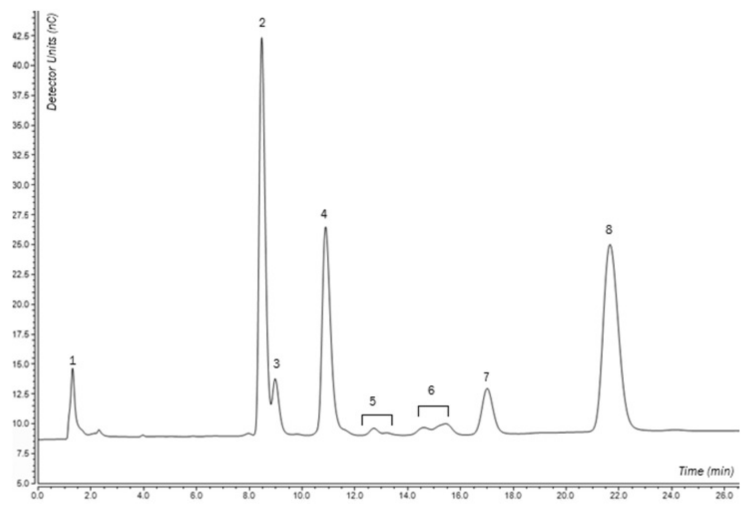


Figure B. High performance anion exchange chromatography trace of CDP liquor.

Figure C. Total ISA concentration in control reactors amended with 50mgml^-1^ chloramphenicol

Figure D. Bradford assayed protein levels across all three systems.

Figure E. Total carbohydrate assay

**Figure F. ISA and ATP assays of the pH 11.0 microcosm.** Measured ISA concentration and relative light units (RLU) in first 12 weeks of sampling. Modelled ISA concentration shown for comparison.

| **Clone ID** | **Closest Sequence Match** | **Similarity** |
| --- | --- | --- |
| 7.5EUB8 | Acidaminobacter hydrogenoformans strain glu 65 16S ribosomal RNA gene, partial sequence | 99 |
| 7.5EUB19 | Acidaminobacter hydrogenoformans strain glu 65 16S ribosomal RNA gene, partial sequence | 99 |
| 7.5EUB37 | Acidaminobacter hydrogenoformans strain glu 65 16S ribosomal RNA gene, partial sequence | 99 |
| 7.5EUB23 | Alkalibacter saccharofermentans strain Z-79820 16S ribosomal RNA gene, partial sequence | 97 |
| 7.5EUB27 | Anaerolinea thermophila strain UNI-1 16S ribosomal RNA gene, complete sequence | 89 |
| 7.5EUB17 | Christensenella minuta strain YIT 12065 16S ribosomal RNA gene, partial sequence | 88 |
| 7.5EUB18 | Christensenella minuta strain YIT 12065 16S ribosomal RNA gene, partial sequence | 88 |
| 7.5EUB26 | Cloacibacillus porcorum strain CL-84 16S ribosomal RNA gene, partial sequence | 91 |
| 7.5EUB48 | Clostridium alkalicellulosi strain Z-7026 | 96 |
| 7.5EUB3 | Clostridium sporosphaeroides strain DSM 1294 16S ribosomal RNA gene, complete sequence | 96 |
| 7.5EUB4 | Clostridium sporosphaeroides strain DSM 1294 16S ribosomal RNA gene, complete sequence | 98 |
| 7.5EUB5 | Clostridium sporosphaeroides strain DSM 1294 16S ribosomal RNA gene, complete sequence | 95 |
| 7.5EUB6 | Clostridium sporosphaeroides strain DSM 1294 16S ribosomal RNA gene, complete sequence | 99 |
| 7.5EUB7 | Clostridium sporosphaeroides strain DSM 1294 16S ribosomal RNA gene, complete sequence | 96 |
| 7.5EUB9 | Clostridium sporosphaeroides strain DSM 1294 16S ribosomal RNA gene, complete sequence | 97 |
| 7.5EUB11 | Clostridium sporosphaeroides strain DSM 1294 16S ribosomal RNA gene, complete sequence | 98 |
| 7.5EUB13 | Clostridium sporosphaeroides strain DSM 1294 16S ribosomal RNA gene, complete sequence | 97 |
| 7.5EUB14 | Clostridium sporosphaeroides strain DSM 1294 16S ribosomal RNA gene, complete sequence | 97 |
| 7.5EUB15 | Clostridium sporosphaeroides strain DSM 1294 16S ribosomal RNA gene, complete sequence | 95 |
| 7.5EUB16 | Clostridium sporosphaeroides strain DSM 1294 16S ribosomal RNA gene, complete sequence | 91 |
| 7.5EUB20 | Clostridium sporosphaeroides strain DSM 1294 16S ribosomal RNA gene, complete sequence | 97 |
| 7.5EUB21 | Clostridium sporosphaeroides strain DSM 1294 16S ribosomal RNA gene, complete sequence | 98 |
| 7.5EUB22 | Clostridium sporosphaeroides strain DSM 1294 16S ribosomal RNA gene, complete sequence | 97 |
| 7.5EUB24 | Clostridium sporosphaeroides strain DSM 1294 16S ribosomal RNA gene, complete sequence | 100 |
| 7.5EUB25 | Clostridium sporosphaeroides strain DSM 1294 16S ribosomal RNA gene, complete sequence | 97 |
| 7.5EUB29 | Clostridium sporosphaeroides strain DSM 1294 16S ribosomal RNA gene, complete sequence | 100 |
| 7.5EUB30 | Clostridium sporosphaeroides strain DSM 1294 16S ribosomal RNA gene, complete sequence | 97 |
| 7.5EUB31 | Clostridium sporosphaeroides strain DSM 1294 16S ribosomal RNA gene, complete sequence | 94 |
| 7.5EUB35 | Clostridium sporosphaeroides strain DSM 1294 16S ribosomal RNA gene, complete sequence | 95 |
| 7.5EUB39 | Clostridium sporosphaeroides strain DSM 1294 16S ribosomal RNA gene, complete sequence | 99 |
| 7.5EUB40 | Clostridium sporosphaeroides strain DSM 1294 16S ribosomal RNA gene, complete sequence | 96 |
| 7.5EUB41 | Clostridium sporosphaeroides strain DSM 1294 16S ribosomal RNA gene, complete sequence | 98 |
| 7.5EUB42 | Clostridium sporosphaeroides strain DSM 1294 16S ribosomal RNA gene, complete sequence | 98 |
| 7.5EUB44 | Clostridium sporosphaeroides strain DSM 1294 16S ribosomal RNA gene, complete sequence | 98 |
| 7.5EUB45 | Clostridium sporosphaeroides strain DSM 1294 16S ribosomal RNA gene, complete sequence | 98 |
| 7.5EUB46 | Clostridium sporosphaeroides strain DSM 1294 16S ribosomal RNA gene, complete sequence | 97 |
| 7.5EUB47 | Draconibacterium orientale 16S ribosomal RNA, complete sequence | 90 |
| 7.5EUB43 | Parabacteroides chartae strain NS31-3 16S ribosomal RNA gene, partial sequence | 88 |
| 7.5EUB28 | Proteiniphilum acetatigenes strain TB107 16S ribosomal RNA gene, complete sequence | 96 |
| 7.5EUB10 | Ruminococcus albus strain 7 16S ribosomal RNA gene, complete sequence | 93 |
| 7.5EUB34 | Saccharofermentans acetigenes strain P6 16S ribosomal RNA gene, complete sequence | 91 |
| 7.5EUB38 | Saccharofermentans acetigenes strain P6 16S ribosomal RNA gene, complete sequence | 93 |
| 7.5EUB12 | Trichococcus pasteurii strain KoTa2 16S ribosomal RNA gene, complete sequence | 99 |
| 7.5EUB32 | Trichococcus pasteurii strain KoTa2 16S ribosomal RNA gene, complete sequence | 99 |
| 7.5EUB33 | Trichococcus pasteurii strain KoTa2 16S ribosomal RNA gene, complete sequence | 98 |
| 7.5EUB36 | Trichococcus pasteurii strain KoTa2 16S ribosomal RNA gene, complete sequence | 99 |
| 7.5EUB1 | Youngiibacter multivorans strain DSM 6139 16S ribosomal RNA gene, partial sequence | 99 |
| 9.5EUB42 | Acetobacterium woodii strain DSM 1030 16S ribosomal RNA gene, complete sequence | 99 |
| 9.5EUB5 | Acidaminobacter hydrogenoformans strain glu 65 16S ribosomal RNA gene, partial sequence | 96 |
| 9.5EUB8 | Acidaminobacter hydrogenoformans strain glu 65 16S ribosomal RNA gene, partial sequence | 97 |
| 9.5EUB11 | Acidaminobacter hydrogenoformans strain glu 65 16S ribosomal RNA gene, partial sequence | 96 |
| 9.5EUB14 | Acidaminobacter hydrogenoformans strain glu 65 16S ribosomal RNA gene, partial sequence | 98 |
| 9.5EUB25 | Acidaminobacter hydrogenoformans strain glu 65 16S ribosomal RNA gene, partial sequence | 96 |
| 9.5EUB45 | Acidaminobacter hydrogenoformans strain glu 65 16S ribosomal RNA gene, partial sequence | 95 |
| 9.5EUB46 | Acidaminobacter hydrogenoformans strain glu 65 16S ribosomal RNA gene, partial sequence | 96 |
| 9.5EUB2 | Alkalibacter saccharofermentans strain Z-79820 16S ribosomal RNA gene, partial sequence | 96 |
| 9.5EUB6 | Alkalibacter saccharofermentans strain Z-79820 16S ribosomal RNA gene, partial sequence | 96 |
| 9.5EUB22 | Alkalibacter saccharofermentans strain Z-79820 16S ribosomal RNA gene, partial sequence | 96 |
| 9.5EUB27 | Alkalibacter saccharofermentans strain Z-79820 16S ribosomal RNA gene, partial sequence | 97 |
| 9.5EUB28 | Alkalibacter saccharofermentans strain Z-79820 16S ribosomal RNA gene, partial sequence | 95 |
| 9.5EUB37 | Alkalibacter saccharofermentans strain Z-79820 16S ribosomal RNA gene, partial sequence | 95 |
| 9.5EUB40 | Alkalibacter saccharofermentans strain Z-79820 16S ribosomal RNA gene, partial sequence | 96 |
| 9.5EUB21 | Aminivibrio pyruvatiphilus strain 4F6E 16S ribosomal RNA gene, partial sequence | 98 |
| 9.5EUB32 | Aminivibrio pyruvatiphilus strain 4F6E 16S ribosomal RNA gene, partial sequence | 97 |
| 9.5EUB17 | Christensenella minuta strain YIT 12065 16S ribosomal RNA gene, partial sequence | 86 |
| 9.5EUB19 | Cloacibacillus porcorum strain CL-84 16S ribosomal RNA gene, partial sequence | 93 |
| 9.5EUB35 | Cloacibacillus porcorum strain CL-84 16S ribosomal RNA gene, partial sequence | 89 |
| 9.5EUB36 | Clostridium thermocellum DSM 1313 16S ribosomal RNA, complete sequence | 90 |
| 9.5EUB29 | Draconibacterium orientale 16S ribosomal RNA, complete sequence | 90 |
| 9.5EUB1 | Paludibacter propionicigenes strain WB4 16S ribosomal RNA gene, complete sequence | 98 |
| 9.5EUB44 | Paludibacter propionicigenes strain WB4 16S ribosomal RNA gene, complete sequence | 89 |
| 9.5EUB31 | Saccharofermentans acetigenes strain P6 16S ribosomal RNA gene, complete sequence | 91 |
| 9.5EUB33 | Saccharofermentans acetigenes strain P6 16S ribosomal RNA gene, complete sequence | 95 |
| 9.5EUB34 | Saccharofermentans acetigenes strain P6 16S ribosomal RNA gene, complete sequence | 95 |
| 9.5EUB13 | Tissierella creatinini strain BN11 16S ribosomal RNA gene, partial sequence | 93 |
| 9.5EUB9 | Trichococcus pasteurii strain KoTa2 16S ribosomal RNA gene, complete sequence | 99 |
| 9.5EUB12 | Trichococcus pasteurii strain KoTa2 16S ribosomal RNA gene, complete sequence | 98 |
| 9.5EUB15 | Trichococcus pasteurii strain KoTa2 16S ribosomal RNA gene, complete sequence | 98 |
| 9.5EUB16 | Trichococcus pasteurii strain KoTa2 16S ribosomal RNA gene, complete sequence | 98 |
| 9.5EUB23 | Trichococcus pasteurii strain KoTa2 16S ribosomal RNA gene, complete sequence | 98 |
| 9.5EUB41 | Trichococcus pasteurii strain KoTa2 16S ribosomal RNA gene, complete sequence | 99 |
| 9.5EUB43 | Trichococcus pasteurii strain KoTa2 16S ribosomal RNA gene, complete sequence | 98 |
| 9.5EUB39 | Youngiibacter fragilis strain 232.1 | 99 |
| 9.5EUB3 | Youngiibacter multivorans strain DSM 6139 16S ribosomal RNA gene, partial sequence | 99 |
| 9.5EUB7 | Youngiibacter multivorans strain DSM 6139 16S ribosomal RNA gene, partial sequence | 98 |
| 9.5EUB10 | Youngiibacter multivorans strain DSM 6139 16S ribosomal RNA gene, partial sequence | 99 |
| 9.5EUB20 | Youngiibacter multivorans strain DSM 6139 16S ribosomal RNA gene, partial sequence | 99 |
| 9.5EUB24 | Youngiibacter multivorans strain DSM 6139 16S ribosomal RNA gene, partial sequence | 87 |
| 9.5EUB30 | Youngiibacter multivorans strain DSM 6139 16S ribosomal RNA gene, partial sequence | 99 |
| 9.5EUB38 | Youngiibacter multivorans strain DSM 6139 16S ribosomal RNA gene, partial sequence | 99 |
| 10EUB1 | Acidaminobacter hydrogenoformans strain glu 65 16S ribosomal RNA gene, partial sequence | 98 |
| 10EUB2 | Acidaminobacter hydrogenoformans strain glu 65 16S ribosomal RNA gene, partial sequence | 97 |
| 10EUB32 | Acidaminobacter hydrogenoformans strain glu 65 16S ribosomal RNA gene, partial sequence | 96 |
| 10EUB33 | Acidaminobacter hydrogenoformans strain glu 65 16S ribosomal RNA gene, partial sequence | 94 |
| 10EUB3 | Alcaligenes aquatilis strain LMG 22996 16S ribosomal RNA gene, partial sequence | 99 |
| 10EUB5 | Alcaligenes aquatilis strain LMG 22996 16S ribosomal RNA gene, partial sequence | 99 |
| 10EUB9 | Alcaligenes aquatilis strain LMG 22996 16S ribosomal RNA gene, partial sequence | 99 |
| 10EUB11 | Alcaligenes aquatilis strain LMG 22996 16S ribosomal RNA gene, partial sequence | 98 |
| 10EUB18 | Alcaligenes aquatilis strain LMG 22996 16S ribosomal RNA gene, partial sequence | 99 |
| 10EUB21 | Alcaligenes aquatilis strain LMG 22996 16S ribosomal RNA gene, partial sequence | 99 |
| 10EUB22 | Alcaligenes aquatilis strain LMG 22996 16S ribosomal RNA gene, partial sequence | 99 |
| 10EUB29 | Alcaligenes aquatilis strain LMG 22996 16S ribosomal RNA gene, partial sequence | 99 |
| 10EUB38 | Alcaligenes aquatilis strain LMG 22996 16S ribosomal RNA gene, partial sequence | 99 |
| 10EUB40 | Alcaligenes aquatilis strain LMG 22996 16S ribosomal RNA gene, partial sequence | 99 |
| 10EUB43 | Alcaligenes aquatilis strain LMG 22996 16S ribosomal RNA gene, partial sequence | 99 |
| 10EUB44 | Alcaligenes aquatilis strain LMG 22996 16S ribosomal RNA gene, partial sequence | 98 |
| 10EUB45 | Alcaligenes aquatilis strain LMG 22996 16S ribosomal RNA gene, partial sequence | 99 |
| 10EUB7 | Alkalibacter saccharofermentans strain Z-79820 16S ribosomal RNA gene, partial sequence | 95 |
| 10EUB28 | Alkalibacter saccharofermentans strain Z-79820 16S ribosomal RNA gene, partial sequence | 96 |
| 10EUB30 | Alkalibacter saccharofermentans strain Z-79820 16S ribosomal RNA gene, partial sequence | 97 |
| 10EUB39 | Alkalibacter saccharofermentans strain Z-79820 16S ribosomal RNA gene, partial sequence | 97 |
| 10EUB26 | Aminivibrio pyruvatiphilus strain 4F6E 16S ribosomal RNA gene, partial sequence | 95 |
| 10EUB8 | Bacillus pseudofirmus OF4 strain OF4 16S ribosomal RNA, complete sequence | 99 |
| 10EUB20 | Cloacibacillus porcorum strain CL-84 16S ribosomal RNA gene, partial sequence | 93 |
| 10EUB37 | Clostridium formicaceticum strain DSM 92 16S ribosomal RNA gene, partial sequence | 96 |
| 10EUB23 | Clostridium thermocellum strain ATCC 27405 16S ribosomal RNA gene, complete sequence | 91 |
| 10EUB24 | Clostridium thermocellum strain ATCC 27405 16S ribosomal RNA gene, complete sequence | 87 |
| 10EUB27 | Clostridium thermocellum strain ATCC 27405 16S ribosomal RNA gene, complete sequence | 88 |
| 10EUB42 | Dehalobacter sp. CF strain CF 16S ribosomal RNA, complete sequence | 94 |
| 10EUB41 | Dehalobacter sp. CF strain CF 16S ribosomal RNA, complete sequence | 93 |
| 10EUB15 | Levilinea saccharolytica strain KIBI-1 16S ribosomal RNA gene, partial sequence | 87 |
| 10EUB31 | Moorella humiferrea strain 64_FGQ | 89 |
| 10EUB34 | Paenibacillus polymyxa strain DSM 36 16S ribosomal RNA gene, partial sequence | 88 |
| 10EUB10 | Saccharofermentans acetigenes strain P6 16S ribosomal RNA gene, complete sequence | 94 |
| 10EUB14 | Saccharofermentans acetigenes strain P6 16S ribosomal RNA gene, complete sequence | 91 |
| 10EUB6 | Sporobacter termitidis strain SYR 16S ribosomal RNA gene, complete sequence | 94 |
| 10EUB12 | Tissierella creatinini strain DSM 9508 16S ribosomal RNA gene, partial sequence | 99 |
| 10EUB4 | Youngiibacter fragilis strain 232.1 16S ribosomal RNA gene, partial sequence | 98 |
| 10EUB36 | Youngiibacter multivorans strain DSM 6139 16S ribosomal RNA gene, partial sequence | 99 |
|  |  |  |
| **Table A. Eubacterial clone libraries of pH 7.5, 9.5 and 10 microcosms, with the closest sequence match within the MEGAblast database** | |  |
|  |  |  |

| **Clone ID** | **Closest Sequence Match** | **Similarity** |
| --- | --- | --- |
| 7.5ARC3 | Methanocorpusculum aggregans strain DSM 3027 16S ribosomal RNA gene, partial sequence | 99 |
| 7.5ARC4 | Methanocorpusculum aggregans strain DSM 3027 16S ribosomal RNA gene, partial sequence | 99 |
| 7.5ARC5 | Methanocorpusculum aggregans strain DSM 3027 16S ribosomal RNA gene, partial sequence | 99 |
| 7.5ARC6 | Methanocorpusculum aggregans strain DSM 3027 16S ribosomal RNA gene, partial sequence | 99 |
| 7.5ARC15 | Methanocorpusculum aggregans strain DSM 3027 16S ribosomal RNA gene, partial sequence | 99 |
| 7.5ARC17 | Methanocorpusculum aggregans strain DSM 3027 16S ribosomal RNA gene, partial sequence | 99 |
| 7.5ARC18 | Methanocorpusculum aggregans strain DSM 3027 16S ribosomal RNA gene, partial sequence | 99 |
| 7.5ARC20 | Methanocorpusculum aggregans strain DSM 3027 16S ribosomal RNA gene, partial sequence | 99 |
| 7.5ARC21 | Methanocorpusculum aggregans strain DSM 3027 16S ribosomal RNA gene, partial sequence | 99 |
| 7.5ARC22 | Methanocorpusculum aggregans strain DSM 3027 16S ribosomal RNA gene, partial sequence | 98 |
| 7.5ARC24 | Methanocorpusculum aggregans strain DSM 3027 16S ribosomal RNA gene, partial sequence | 99 |
| 7.5ARC26 | Methanocorpusculum aggregans strain DSM 3027 16S ribosomal RNA gene, partial sequence | 99 |
| 7.5ARC28 | Methanocorpusculum aggregans strain DSM 3027 16S ribosomal RNA gene, partial sequence | 99 |
| 7.5ARC31 | Methanocorpusculum aggregans strain DSM 3027 16S ribosomal RNA gene, partial sequence | 99 |
| 7.5ARC33 | Methanocorpusculum aggregans strain DSM 3027 16S ribosomal RNA gene, partial sequence | 99 |
| 7.5ARC34 | Methanocorpusculum aggregans strain DSM 3027 16S ribosomal RNA gene, partial sequence | 99 |
| 7.5ARC36 | Methanocorpusculum aggregans strain DSM 3027 16S ribosomal RNA gene, partial sequence | 99 |
| 7.5ARC37 | Methanocorpusculum aggregans strain DSM 3027 16S ribosomal RNA gene, partial sequence | 99 |
| 7.5ARC38 | Methanocorpusculum aggregans strain DSM 3027 16S ribosomal RNA gene, partial sequence | 99 |
| 7.5ARC39 | Methanocorpusculum aggregans strain DSM 3027 16S ribosomal RNA gene, partial sequence | 99 |
| 7.5ARC43 | Methanocorpusculum aggregans strain DSM 3027 16S ribosomal RNA gene, partial sequence | 99 |
| 7.5ARC44 | Methanocorpusculum aggregans strain DSM 3027 16S ribosomal RNA gene, partial sequence | 99 |
| 7.5ARC47 | Methanocorpusculum aggregans strain DSM 3027 16S ribosomal RNA gene, partial sequence | 99 |
| 7.5ARC16 | Methanocorpusculum aggregans strain DSM 3027 16S ribosomal RNA gene, partial sequence | 98 |
| 7.5ARC8 | Methanocorpusculum labreanum strain Z 16S ribosomal RNA gene, complete sequence | 99 |
| 7.5ARC9 | Methanomassiliicoccus luminyensis strain B10 16S ribosomal RNA gene, partial sequence | 99 |
| 7.5ARC11 | Methanomassiliicoccus luminyensis strain B10 16S ribosomal RNA gene, partial sequence | 94 |
| 7.5ARC13 | Methanomassiliicoccus luminyensis strain B10 16S ribosomal RNA gene, partial sequence | 94 |
| 7.5ARC30 | Methanosaeta concilii strain GP6 16S ribosomal RNA gene, complete sequence | 95 |
| 7.5ARC27 | Methanosarcina lacustris strain ZS 16S ribosomal RNA gene, partial sequence | 99 |
| 7.5ARC2 | Methanosarcina siciliae strain T4/M 16S ribosomal RNA gene, partial sequence | 99 |
| 7.5ARC10 | Methanosarcina siciliae strain T4/M 16S ribosomal RNA gene, partial sequence | 99 |
| 7.5ARC32 | Methanosarcina siciliae strain T4/M 16S ribosomal RNA gene, partial sequence | 99 |
| 7.5ARC35 | Methanosarcina siciliae strain T4/M 16S ribosomal RNA gene, partial sequence | 99 |
| 7.5ARC46 | Methanosarcina siciliae strain T4/M 16S ribosomal RNA gene, partial sequence | 99 |
| 7.5ARC25 | Methanosarcina vacuolata strain Z-761 16S ribosomal RNA gene, partial sequence | 99 |
| 7.5ARC7 | Methanosphaerula palustris strain E1-9c 16S ribosomal RNA gene, complete sequence | 91 |
| 7.5ARC14 | Methanosphaerula palustris strain E1-9c 16S ribosomal RNA gene, complete sequence | 96 |
| 7.5ARC19 | Methanosphaerula palustris strain E1-9c 16S ribosomal RNA gene, complete sequence | 95 |
| 7.5ARC23 | Methanosphaerula palustris strain E1-9c 16S ribosomal RNA gene, complete sequence | 94 |
| 7.5ARC29 | Methanosphaerula palustris strain E1-9c 16S ribosomal RNA gene, complete sequence | 94 |
| 7.5ARC1 | Thermofilum pendens Hrk 5 16S ribosomal RNA, complete sequence | 84 |
| 7.5ARC12 | Thermofilum pendens Hrk 5 16S ribosomal RNA, complete sequence | 83 |
| 7.5ARC40 | Thermofilum pendens Hrk 5 16S ribosomal RNA, complete sequence | 84 |
| 7.5ARC41 | Thermofilum pendens Hrk 5 16S ribosomal RNA, complete sequence | 84 |
| 9.5ARC4 | Methanobacterium alcaliphilum strain NBRC 105226 16S ribosomal RNA gene, partial sequence | 99 |
| 9.5ARC2 | Methanobacterium flexile strain GH 16S ribosomal RNA gene, partial sequence | 99 |
| 9.5ARC12 | Methanobacterium flexile strain GH 16S ribosomal RNA gene, partial sequence | 99 |
| 9.5ARC13 | Methanobacterium flexile strain GH 16S ribosomal RNA gene, partial sequence | 99 |
| 9.5ARC15 | Methanobacterium flexile strain GH 16S ribosomal RNA gene, partial sequence | 99 |
| 9.5ARC16 | Methanobacterium flexile strain GH 16S ribosomal RNA gene, partial sequence | 99 |
| 9.5ARC17 | Methanobacterium flexile strain GH 16S ribosomal RNA gene, partial sequence | 99 |
| 9.5ARC18 | Methanobacterium flexile strain GH 16S ribosomal RNA gene, partial sequence | 99 |
| 9.5ARC19 | Methanobacterium flexile strain GH 16S ribosomal RNA gene, partial sequence | 99 |
| 9.5ARC20 | Methanobacterium flexile strain GH 16S ribosomal RNA gene, partial sequence | 99 |
| 9.5ARC28 | Methanobacterium flexile strain GH 16S ribosomal RNA gene, partial sequence | 99 |
| 9.5ARC29 | Methanobacterium flexile strain GH 16S ribosomal RNA gene, partial sequence | 99 |
| 9.5ARC30 | Methanobacterium flexile strain GH 16S ribosomal RNA gene, partial sequence | 99 |
| 9.5ARC32 | Methanobacterium flexile strain GH 16S ribosomal RNA gene, partial sequence | 99 |
| 9.5ARC34 | Methanobacterium flexile strain GH 16S ribosomal RNA gene, partial sequence | 99 |
| 9.5ARC37 | Methanobacterium flexile strain GH 16S ribosomal RNA gene, partial sequence | 99 |
| 9.5ARC40 | Methanobacterium flexile strain GH 16S ribosomal RNA gene, partial sequence | 99 |
| 9.5ARC47 | Methanobacterium flexile strain GH 16S ribosomal RNA gene, partial sequence | 99 |
| 9.5ARC42 | Methanobacterium palustre strain F 16S ribosomal RNA gene, partial sequence | 99 |
| 9.5ARC7 | Methanobacterium subterraneum strain A8p 16S ribosomal RNA gene, partial sequence | 99 |
| 9.5ARC27 | Methanobacterium subterraneum strain A8p 16S ribosomal RNA gene, partial sequence | 99 |
| 9.5ARC38 | Methanobacterium subterraneum strain A8p 16S ribosomal RNA gene, partial sequence | 99 |
| 9.5ARC5 | Methanocorpusculum aggregans strain DSM 3027 16S ribosomal RNA gene, partial sequence | 99 |
| 9.5ARC39 | Methanomassiliicoccus luminyensis strain B10 16S ribosomal RNA gene, partial sequence | 89 |
| 9.5ARC1 | Methanomassiliicoccus luminyensis strain B10 16S ribosomal RNA gene, partial sequence | 89 |
| 9.5ARC3 | Methanomassiliicoccus luminyensis strain B10 16S ribosomal RNA gene, partial sequence | 88 |
| 9.5ARC6 | Methanomassiliicoccus luminyensis strain B10 16S ribosomal RNA gene, partial sequence | 89 |
| 9.5ARC8 | Methanomassiliicoccus luminyensis strain B10 16S ribosomal RNA gene, partial sequence | 89 |
| 9.5ARC9 | Methanomassiliicoccus luminyensis strain B10 16S ribosomal RNA gene, partial sequence | 89 |
| 9.5ARC10 | Methanomassiliicoccus luminyensis strain B10 16S ribosomal RNA gene, partial sequence | 88 |
| 9.5ARC14 | Methanomassiliicoccus luminyensis strain B10 16S ribosomal RNA gene, partial sequence | 89 |
| 9.5ARC23 | Methanomassiliicoccus luminyensis strain B10 16S ribosomal RNA gene, partial sequence | 88 |
| 9.5ARC24 | Methanomassiliicoccus luminyensis strain B10 16S ribosomal RNA gene, partial sequence | 89 |
| 9.5ARC25 | Methanomassiliicoccus luminyensis strain B10 16S ribosomal RNA gene, partial sequence | 88 |
| 9.5ARC26 | Methanomassiliicoccus luminyensis strain B10 16S ribosomal RNA gene, partial sequence | 88 |
| 9.5ARC31 | Methanomassiliicoccus luminyensis strain B10 16S ribosomal RNA gene, partial sequence | 88 |
| 9.5ARC33 | Methanomassiliicoccus luminyensis strain B10 16S ribosomal RNA gene, partial sequence | 88 |
| 9.5ARC35 | Methanomassiliicoccus luminyensis strain B10 16S ribosomal RNA gene, partial sequence | 89 |
| 9.5ARC36 | Methanomassiliicoccus luminyensis strain B10 16S ribosomal RNA gene, partial sequence | 89 |
| 9.5ARC41 | Methanomassiliicoccus luminyensis strain B10 16S ribosomal RNA gene, partial sequence | 89 |
| 9.5ARC43 | Methanomassiliicoccus luminyensis strain B10 16S ribosomal RNA gene, partial sequence | 89 |
| 9.5ARC44 | Methanomassiliicoccus luminyensis strain B10 16S ribosomal RNA gene, partial sequence | 89 |
| 9.5ARC45 | Methanomassiliicoccus luminyensis strain B10 16S ribosomal RNA gene, partial sequence | 88 |
| 9.5ARC46 | Methanomassiliicoccus luminyensis strain B10 16S ribosomal RNA gene, partial sequence | 89 |
| 9.5ARC48 | Methanomassiliicoccus luminyensis strain B10 16S ribosomal RNA gene, partial sequence | 89 |
| 9.5ARC11 | Methanosphaerula palustris strain E1-9c 16S ribosomal RNA gene, complete sequence | 94 |
| 9.5ARC21 | Methanosphaerula palustris strain E1-9c 16S ribosomal RNA gene, complete sequence | 95 |
| 9.5ARC22 | Methanosphaerula palustris strain E1-9c 16S ribosomal RNA gene, complete sequence | 96 |
| 10ARC23 | Methanobacterium alcaliphilum strain NBRC 105226 16S ribosomal RNA gene, partial sequence | 99 |
| 10ARC24 | Methanobacterium alcaliphilum strain NBRC 105226 16S ribosomal RNA gene, partial sequence | 99 |
| 10ARC35 | Methanobacterium alcaliphilum strain NBRC 105226 16S ribosomal RNA gene, partial sequence | 99 |
| 10ARC7 | Methanobacterium flexile strain GH 16S ribosomal RNA gene, partial sequence | 99 |
| 10ARC18 | Methanobacterium flexile strain GH 16S ribosomal RNA gene, partial sequence | 99 |
| 10ARC21 | Methanobacterium flexile strain GH 16S ribosomal RNA gene, partial sequence | 99 |
| 10ARC38 | Methanobacterium flexile strain GH 16S ribosomal RNA gene, partial sequence | 99 |
| 10ARC40 | Methanobacterium flexile strain GH 16S ribosomal RNA gene, partial sequence | 99 |
| 10ARC5 | Methanobacterium subterraneum strain A8p 16S ribosomal RNA gene, partial sequence | 99 |
| 10ARC32 | Methanobacterium subterraneum strain A8p 16S ribosomal RNA gene, partial sequence | 99 |
| 10ARC45 | Methanobacterium subterraneum strain A8p 16S ribosomal RNA gene, partial sequence | 99 |
| 10ARC47 | Methanobacterium subterraneum strain A8p 16S ribosomal RNA gene, partial sequence | 99 |
| 10ARC11 | Methanocalculus taiwanensis strain P2F9704a 16S ribosomal RNA gene, partial sequence | 99 |
| 10ARC25 | Methanocalculus taiwanensis strain P2F9704a 16S ribosomal RNA gene, partial sequence | 99 |
| 10ARC1 | Methanocorpusculum aggregans strain DSM 3027 16S ribosomal RNA gene, partial sequence | 99 |
| 10ARC3 | Methanocorpusculum aggregans strain DSM 3027 16S ribosomal RNA gene, partial sequence | 99 |
| 10ARC4 | Methanocorpusculum aggregans strain DSM 3027 16S ribosomal RNA gene, partial sequence | 99 |
| 10ARC6 | Methanocorpusculum aggregans strain DSM 3027 16S ribosomal RNA gene, partial sequence | 99 |
| 10ARC8 | Methanocorpusculum aggregans strain DSM 3027 16S ribosomal RNA gene, partial sequence | 99 |
| 10ARC10 | Methanocorpusculum aggregans strain DSM 3027 16S ribosomal RNA gene, partial sequence | 99 |
| 10ARC12 | Methanocorpusculum aggregans strain DSM 3027 16S ribosomal RNA gene, partial sequence | 99 |
| 10ARC13 | Methanocorpusculum aggregans strain DSM 3027 16S ribosomal RNA gene, partial sequence | 99 |
| 10ARC14 | Methanocorpusculum aggregans strain DSM 3027 16S ribosomal RNA gene, partial sequence | 99 |
| 10ARC15 | Methanocorpusculum aggregans strain DSM 3027 16S ribosomal RNA gene, partial sequence | 99 |
| 10ARC16 | Methanocorpusculum aggregans strain DSM 3027 16S ribosomal RNA gene, partial sequence | 99 |
| 10ARC17 | Methanocorpusculum aggregans strain DSM 3027 16S ribosomal RNA gene, partial sequence | 99 |
| 10ARC26 | Methanocorpusculum aggregans strain DSM 3027 16S ribosomal RNA gene, partial sequence | 99 |
| 10ARC27 | Methanocorpusculum aggregans strain DSM 3027 16S ribosomal RNA gene, partial sequence | 99 |
| 10ARC29 | Methanocorpusculum aggregans strain DSM 3027 16S ribosomal RNA gene, partial sequence | 99 |
| 10ARC30 | Methanocorpusculum aggregans strain DSM 3027 16S ribosomal RNA gene, partial sequence | 99 |
| 10ARC33 | Methanocorpusculum aggregans strain DSM 3027 16S ribosomal RNA gene, partial sequence | 99 |
| 10ARC41 | Methanocorpusculum aggregans strain DSM 3027 16S ribosomal RNA gene, partial sequence | 99 |
| 10ARC43 | Methanocorpusculum aggregans strain DSM 3027 16S ribosomal RNA gene, partial sequence | 99 |
| 10ARC46 | Methanocorpusculum aggregans strain DSM 3027 16S ribosomal RNA gene, partial sequence | 99 |
| 10ARC9 | Methanosarcina mazei Go1 16S ribosomal RNA, complete sequence | 99 |
| 10ARC20 | Methanosarcina siciliae strain T4/M 16S ribosomal RNA gene, partial sequence | 99 |
| 10ARC34 | Methanosarcina siciliae strain T4/M 16S ribosomal RNA gene, partial sequence | 99 |
| 10ARC36 | Methanosarcina siciliae strain T4/M 16S ribosomal RNA gene, partial sequence | 99 |
| 10ARC19 | Methanosarcina vacuolata strain Z-761 16S ribosomal RNA gene, partial sequence | 99 |
| 10ARC2 | Methanosphaerula palustris strain E1-9c 16S ribosomal RNA gene, complete sequence | 95 |
| 10ARC28 | Methanosphaerula palustris strain E1-9c 16S ribosomal RNA gene, complete sequence | 95 |
| 10ARC31 | Methanosphaerula palustris strain E1-9c 16S ribosomal RNA gene, complete sequence | 95 |
| 10ARC37 | Methanosphaerula palustris strain E1-9c 16S ribosomal RNA gene, complete sequence | 95 |
| 10ARC44 | Methanosphaerula palustris strain E1-9c 16S ribosomal RNA gene, complete sequence | 95 |
| 10ARC22 | Thermofilum pendens Hrk 5 16S ribosomal RNA, complete sequence | 84 |
|  |  |  |
| **Table B. Archaeal clone libraries of pH 7.5, 9.5 and 10 microcosms, with the closest sequence match within the MEGAblast database** | |  |
|  |  |  |
|  |  |  |
